# On the determination of elastic moduli of cells by AFM based indentation

**DOI:** 10.1038/srep45575

**Published:** 2017-04-03

**Authors:** Yue Ding, Guang-Kui Xu, Gang-Feng Wang

**Affiliations:** 1 Department of Engineering Mechanics, State Key Laboratory for Strength and Vibration of Mechanical Structures, Xi’an Jiaotong University, Xi’an 710049, China; 2 International Center for Applied Mechanics, State Key Laboratory for Strength and Vibration of Mechanical Structures, Xi’an Jiaotong University, Xi’an 710049, China

## Abstract

The atomic force microscopy (AFM) has been widely used to measure the mechanical properties of biological cells through indentations. In most of existing studies, the cell is supposed to be linear elastic within the small strain regime when analyzing the AFM indentation data. However, in experimental situations, the roles of large deformation and surface tension of cells should be taken into consideration. Here, we use the neo-Hookean model to describe the hyperelastic behavior of cells and investigate the influence of surface tension through finite element simulations. At large deformation, a correction factor, depending on the geometric ratio of indenter radius to cell radius, is introduced to modify the force-indent depth relation of classical Hertzian model. Moreover, when the indent depth is comparable with an intrinsic length defined as the ratio of surface tension to elastic modulus, the surface tension evidently affects the indentation response, indicating an overestimation of elastic modulus by the Hertzian model. The dimensionless-analysis-based theoretical predictions, which include both large deformation and surface tension, are in good agreement with our finite element simulation data. This study provides a novel method to more accurately measure the mechanical properties of biological cells and soft materials in AFM indentation experiments.

Studies of the mechanics of biological cells are crucial for understanding a variety of fundamental cell behaviors, such as motility^1^, differentiation^2^ and proliferation^3^, and have attracted tremendous attention in the fields of tissue engineering, cell biology and cancer treatment^4^,^5^. To measure the mechanical properties of cells, various experimental techniques, such as optical stretcher^6^, micropipette aspiration^7^ and magnetic twisting cytometry^8^, have been developed. Among them, atomic force microscopy (AFM), first developed as a surface imaging tool in 1986^9^, has become the most popular and useful tool to characterize the mechanics of diseased and healthy cells at different stages of the cell-cycle^10^,^11^,^12^.

By using AFM, considerable efforts have been directed towards measuring the mechanical properties of various types of cells. For example, by using the AFM-based single-cell compression, Lulevich *et al*. measured the load-depth curves of living and dead cells and revealed the different cellular mechanical responses^13^. Nguyen and Gu^14^ demonstrated that the Young’s moduli of chondrocytes are strain-rate-dependent, similar to the viscoelastic features detected in alveolar and bronchial cells^15^. The diabetes- and aging-induced changes in morphological and mechanical properties were also studied by using AFM^16^. Furthermore, different types of cancer cells have been measured^17^,^18^,^19^, and are found to be up to 70% softer than the benign cells^4^. Usually, in the AFM experiments, the elastic moduli of cells are obtained through fitting the force-displacement curves by using Hertzian model^20^ for spherical indenter or Sneddon’s model^21^ for conical indenter. By treating the cell as an elastic layer with finite thickness, Dimitriadis *et al*.^22^ and Gavara and Chadwick^23^ formulated the corresponding solutions for spherical and conical indenters, respectively. In most of these existing studies, the cell is supposed to be linear elastic material and its deformation obeys the small strain assumption. However, in fact, cells usually undergo large deformation in the indentation experiments^13^,^14^,^15^ and the existing models do not agree well with experimental situations^24^. Therefore, it is necessary to modify the traditional contact model by accounting for cellular large deformation in the AFM indentation experiments.

Moreover, surface tension has been proved to regulate the shape of cells^25^,^26^ and significantly affect the envelopment behavior of biological cells and tissues^27^. For soft materials (e.g., PDMS and hydrogel), surface tension can also affect their geometric shape^28^ and stability^29^, as well as the contact mechanism^30^. For linear elastic material, the influences of surface effects on contact problem have been theoretically investigated. For instance, Hajji analyzed the indentation on an elastic half space with surface tension to measure the deformation of the inflated lobes^31^. Long and Wang studied the effect of surface tension on the elastic field of an elastic half space indented by a rigid sphere^32^. Gao *et al*. found that the residual surface tension plays a dominant role over surface elasticity in the spherical indentation^33^. Xu *et al*. investigated the role of surface tension in the adhesion between a rigid spherical indenter and a hyperelastic soft material^34^. However, to our knowledge, there are still no studies to examine the effect of the cellular surface tension on measuring elastic stiffness in AFM experiments.

In this paper, we develop a novel method to consider both large deformation and surface tension when measuring the elastic moduli of cells in the AFM indentation experiments. Based on the dimensional analysis and finite element simulations, explicit expressions of load-depth curves are achieved for various size ratios between the indenter and the cell. We demonstrate that at large deformation, a correction factor, relating to the geometric ratio of indenter radius to cell radius, should be introduced to modify the Hertzian solution. More importantly, surface tension will remarkably affect the indentation response, indicating an overestimation of elastic modulus by classical Hertzian model. Our theoretical results agree well with the finite element simulation data. This method provides a more accurate avenue to measure the elastic modulus of cells in the AFM indentation experiments.

## Finite Element Simulations

**Figure 1:**
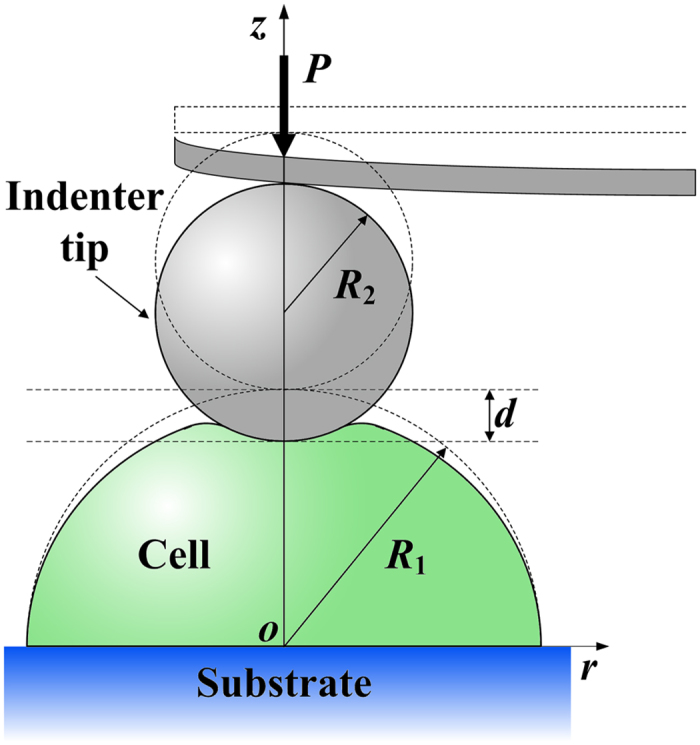
Schematic of indentation on an isolated hemispherical cell. The cell with radius *R*_1_ is located on a rigid flat substrate and indented by a spherical indenter with radius *R*_2_. For convenience, a cylindrical coordinate system with the origin at the center of the cell and the *z*-axis along the compressive direction is established.

Consider that we measure the elastic modulus of a cell placing on a substrate by using AFM indentation methods. For simplicity, we study a hemispherical cell with radius *R*_1_ indented by a rigid spherical indenter with radius *R*_2_, as illustrated in Fig. 1. It is straightforward to model other cell shapes (e.g., spherical crown and thin sheet) using the proposed method. The external load *P* is applied on the cell through the spherical indenter, leading to an indent depth *d*. The finite element simulations are performed using the commercial finite element methods (FEM) software, ABAQUS.

In our finite element simulations, the cell is described as an incompressible hyperelastic material obeying the neo-Hookean constitutive law, which has been successfully used to study the mechanical response of cells^35^,^36^. The strain energy density *U* of the neo-Hookean model is given by


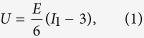


where *E* is the Young’s modulus and 
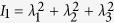
 is the first invariant of the principal stretches *λ*_*i*_. A constant surface energy density is assumed on the cell surface, which corresponds also a constant residual surface tension^37^. The effect of surface energy is introduced into ABAQUS by surface elements via the user subroutine UEL. To solve this nonlinear problem, Newton-Raphson method in ABAQUS is employed to seek the finial equilibrium state. The details of FEM simulations with the incorporation of surface energy can be found in our previous work^37^.

The experiments showed that the elastic modulus *E* of cells is in the range of 0.1–100 kPa^10^,^23^,^38^, and here we take *E* = 1 kPa in our simulations. The cellular radius is taken as *R*_1_ = 10 μm^39^, and the surface energy density *γ* is varied from 0 to 0.05 N/m^40^. The radius *R*_2_ of the spherical indenter is varied from 1 μm to 100 μm^13^,^14^,^17^, which leads the geometric size ratio *β* = *R*_2_/*R*_1_ to be in the range of 0.1–10. The cell is meshed with 4-node bilinear axisymmetric quadrilateral hybrid reduced integration elements and user-defined elements considering surface energy are defined to be attached on the surface. The spherical indenter is treated to be rigid, and the contact between the indenter and the cell is assumed to be frictionless. For smaller indenters (*β* < 1), the maximum indent depth is set as *d* = 0.3*R*_2_, while for larger indenters (*β *>* *1), the limit of the indent depth is *d* = 0.3*R*_1_. In all cases, convergence tests have been carried out to ensure the accuracy of computational results.

## Results and Discussion

### Indentation on cells at large deformation without surface energy

Firstly, we study the load-indent depth relation of cells at large deformation without considering the cellular surface energy. For an elastic sphere with radius *R*_1_ compressed by a rigid spherical indenter with radius *R*_2_, the classical Hertzian contact theory, based on the small strain assumption, gives the relation^20^


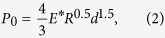


where *P*_0_ is the load, *d* is the indent depth, E* = *E*/(1 − *v*^2^) is the combined elastic modulus with *E* and *v* being the Young’s modulus and Poisson’s ratio, and *R* is the equivalent radius given by 1/*R* = 1/*R*_1_ + 1/*R*_2_. However, when an isolated cell is indented at large deformation, the linear elastic assumption in Hertzian theory is not valid, and the hyperelastic behavior should be taken into account.

**Figure 2:**
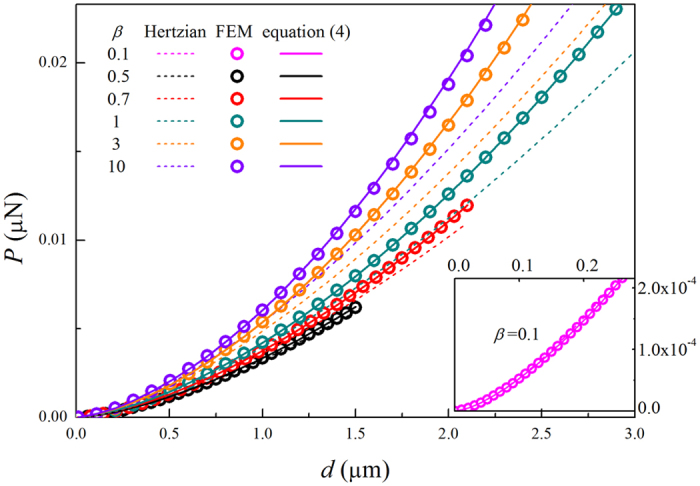
Load-depth curves for different indenter radii. For small ratio *β* = 0.1, an inset is shown in this figure. The symbols are calculated by FEM simulations, and can be well fitted by equation (4) (see solid lines). The dashed lines represent the classic Hertzian solutions by using equation (2).

In the case of large deformation, we use the neo-Hookean constitutive law to describe the hyperelastic property of cells. According to the dimensional analysis^41^, the load *P*_nH_ can be expressed as


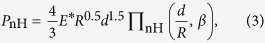


where П_nH_ is a dimensionless function depending on the size ratio *β* and the ratio of the indent depth *d* to the equivalent radius *R*. Using FEM, we calculate the load *P*_nH_ as a function of the indentation depth *d* for different ratios (*β* = *R*_2_/*R*_1_) from 0.1 to 10, as shown in Fig. 2. For comparison, we also plot the prediction of classical Hertzian model by using equation (2). Interestingly, we found that for all ratios *β*, the load-indentation FEM data can be fitted very well by the same function


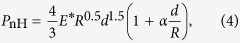


where *α* represents a fitting parameter depending on the ratio *β*. Different from the Hertzian solution, the above equation includes the contributions from *d*/*R* and *α* for large deformation, whose roles are described as follows.

For small indent depths (e.g., *d*/*R*<<1), the FEM data coincides with the Hertzian solutions, as shown in Fig. 2. However, for larger indent depths, the influence of large deformation becomes important, especially when the geometric ratio *β* is large. As the ratio of the indent depth *d* to the equivalent radius *R* increases, the difference between FEM data and Hertzian predictions gets significant, and thus it is necessary to employ equation (4) to characterize the compressive response. Equation (4) provides a clue to examine the effect of large deformation in the AFM based indentation, by indenting the cell at different depths and calculating the relevant Young’s moduli.

**Figure 3:**
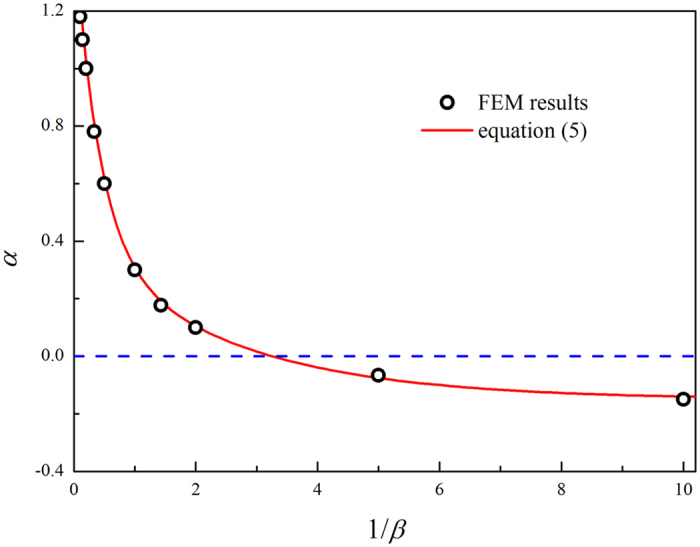
Values of *α* for different ratios *β* of indenter radius to cell radius. The symbols represent the fitting parameter obtained from equation (4). The solid line is plotted by equation (5), which is in good agreement with the symbols.

The dependence of *α* on the size ratio *β* is calculated, as displayed in Fig. 3. It can be seen that the data can be fitted well by


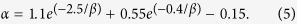


The curve can be divided into two regimes, according to the sign of the parameter *α*. A positive or negative value of *α* indicates the overestimation or underestimation of the Hertzian model. Figure 3 shows that the critical indenter radius is *R*_2c_ = 0.3*R*_1_ (i.e., *β* = 0.3). When the indenter is small (e.g., *β* < 0.3), *α* is a bit smaller than zero, and the elastic modulus would be slightly underestimated by Hertzian theory. For large indenters (e.g., *β *>* *0.3), the actual force acting on the cell will be larger than the Hertzian prediction (Fig. 2), which leads to an overestimation of the elastic modulus using Hertzian model. Furthermore, the parameter *α* increases with increasing *β*. The bigger the indenter radius *R*_2_ is, thus the larger the geometric ratio *β* is, then the more significant the difference between equation (4) and Hertzian prediction is. When the indenter size is much larger than the cell size (*β* >>1), equation (4) reduces to the case for the compression of cell by a rigid plane, which has been investigated previously^42^. It is interesting to notice that, when *β* is close to the critical size ratio 0.3, equation (4) approaches to Hertzian solution, which gives an implication to design the indenter radius to avoid the influence of large deformation.

### Indentation on cells at large deformation with surface energy

**Figure 4:**
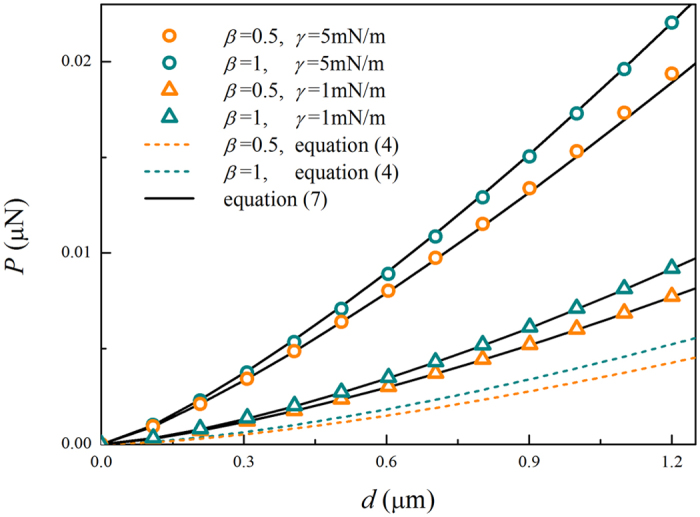
Load-depth curves affected by the surface energy for two indenter radii. The symbols are from the FEM results with considering surface energy and can be predicted by equation (7) (see solid lines). The dashed lines represent the results without surface energy obtained from equation (4).

Then, we investigate the effect of surface energy on the AFM indentation measurement of cells. For two size ratios (*β* = 0.5 and *β* = 1), Fig. 4 plots the load with respect to the indent depth under two representative values of surface energy (*γ* = 1 mN/m and *γ* = 5 mN/m). The results from equation (4) for the case without surface energy are also included for comparison. It can be clearly seen that surface energy can evidently affect the load-depth curve. For a given value of the indent depth *d*, the load considering surface energy is much larger than that neglecting surface energy. For example, when the sizes of the indenter and the cell are the same (i.e., *β* = 1), the load with surface energy (*γ* = 5 mN/m) can be three times larger than that without surface effect at *d* = 1.2 μm. Further, to generate a given indent depth, a larger load will be required as the surface energy density increases.

To achieve the analytical relation of load-depth for the compression of hyperelastic cell with surface energy under large deformation, the dimensional analysis approach is adopted to analyze numerous FEM data under different indenter sizes. The intrinsic length that surface energy affects in solids can be indicated by the ratio of surface energy to elastic modulus, as 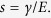 Then, according to dimensional analysis, the indentation load *P*_nHs_ should be a function of the following independent parameters


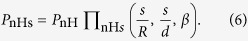


where *P*_nH_ is the force without considering surface energy given by equation (4), and П_nHs_ is a dimensionless function of three terms: *s*/*R, s*/*d* and *β*.

**Figure 5:**
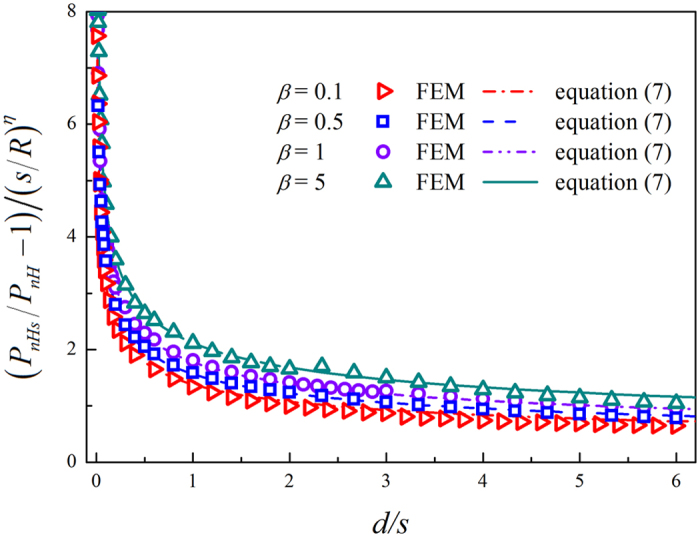
Relation between the normalized load and the normalized indent depth. The symbols are from finite element simulations, and the lines represent the predictions of equation (7).

For *d* ≤ 0.2*R*_1_, Fig. 5 shows the normalized load with respect to the normalized indent depth *d*/*s* under several ratios *β*. It can be seen that when the indent depth *d* is much larger than the intrinsic length *s*, the contribution of surface energy is negligible, as found in our previous work^42^. However, when the indent depth is comparable with the intrinsic length or even smaller, surface energy will significantly affect the load-indent depth relation.

The dependence of load on indent depth can be well described by


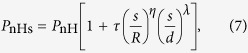


where *τ, η* and *λ* are three parameters depending on the geometric ratio *β*. We conduct numerical calculations for various ratios of *β* (e.g., *β* = 0.1, 0.3, 0.5, 0.7, 1, 3, 5, 7 and 10), and find that these parameters can be fitted by


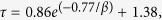



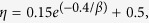



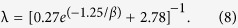


The predictions of equation (7) are displayed in Figs 4 and 5, which show excellent agreement with FEM data. For a large size of indenter (*β* >> 1), equation (7) reduces to the result of the compression of a hyperelastic cell with surface energy by a rigid plane^42^, which validates the present results to some extent.

Therefore, equation (7) characterizes well the overall compressive response of hyperelastic cell under large deformation, and accounts for both the influence of surface energy and the shape of indenters. For cells with larger surface energy, the modification from equation (7) to the Hertzian prediction is much significant, and equation (7) should be employed to extract the elastic modulus from the load-depth curve. Otherwise, the direct application of Hertzian model could remarkably overestimate the elastic modulus of cells. Since the surface tension can be regulated by using drugs, its role could be examined in the AFM indentation experiments.

### Indentation on spherical cells with surface energy.

**Figure 6:**
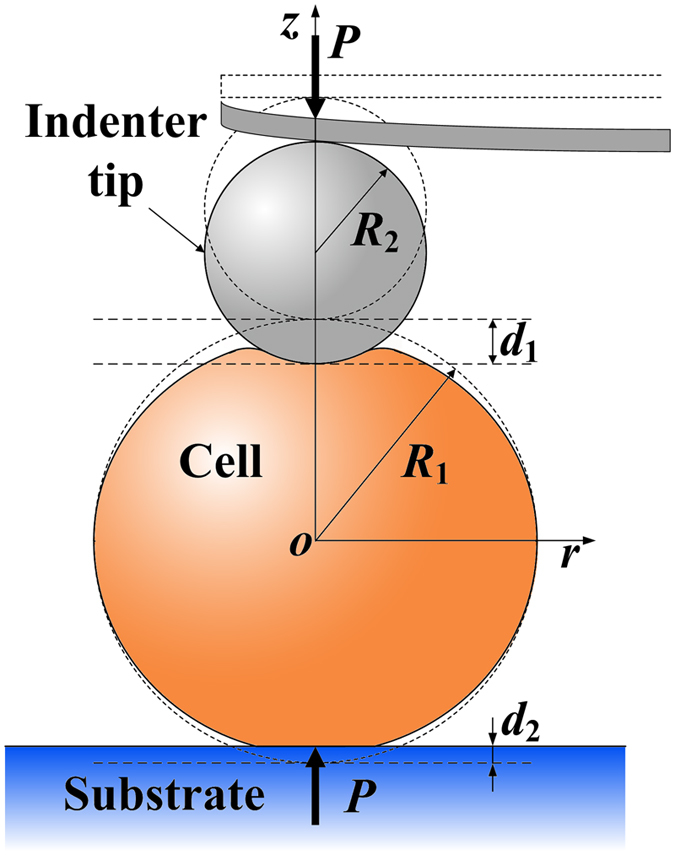
Schematic of indentation on a spherical cell. The spherical cell with radius *R*_1_ is placed on the rigid plane and indented by a rigid spherical indenter with radius *R*_2_ from the upper side. The indent depth from upper indentation is *d*_1_ and from bottom compression is *d*_2_.

In some experimental circumstances, the biological cell takes a roughly spherical shape, and the AFM indentation is conducted on the top side, as schematically shown in Fig. 6. We further extend our model to this practical case. In this loading scenario, the indent depth *d* measured by the indenter of AFM is the sum of the indent depth *d*_1_ from the top indentation by the spherical AFM indenter, and the indent depth *d*_2_ from the bottom by planar compression, that is,


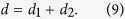


The indent depth *d*_1_ is already given by equation (7). And according to our previous analysis^42^, *d*_2_ is related to the compressive load by


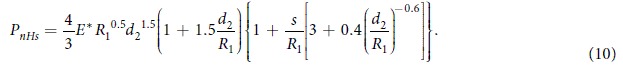


Combining equations (7), (9) and (10), one can obtain the analytical expressions of the load-indent depth relation for this case.

If surface energy is excluded, the top and bottom indent depth *d*_1_ and *d*_2_ depend on the load as


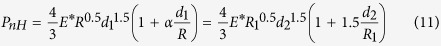


Substituting equation (11) into equation (9) gives the analytical solution of this contact problem with vanishing surface energy.

## Conclusions

The AFM indentation on hyperelastic cells with surface tension has been investigated through finite element method in this work. When the indent depth is close to the equivalent radius, the large deformation should be taken into account, since it can remarkably alter the force-indent depth relation of the classical Hertzian solution. The FEM results indicate that Hertzian model would overestimate the elastic modulus of cell when the ratio of indenter radius to cell radius is larger than 0.3, but underestimate the modulus when the ratio is less than 0.3. When the indent depth is comparable with the intrinsic length, the surface tension greatly affects the indentation response, indicating that elastic modulus would be markedly overestimated by the classical Hertzian model. By performing dimensional analysis, we present the analytical expressions between the load and indent depth, which takes into account both large deformation and surface tension. The theoretical predictions are in good agreement with our FEM data. In addition, the analytical solution for an indenter on a spherical cell placing on a flat rigid plane is also provided in the paper. It is straightforward to extend the present method to other cell shapes, which will be addressed in the future. These results are beneficial for more accurately and correctly calculating the elastic modulus of cells in the AFM indentation experiments.

## Additional Information

**How to cite this article**: Ding, Y. *et al*. On the determination of elastic moduli of cells by AFM based indentation. *Sci. Rep.* **7**, 45575; doi: 10.1038/srep45575 (2017).

**Publisher's note:** Springer Nature remains neutral with regard to jurisdictional claims in published maps and institutional affiliations.
